# Risk-reducing salpingo-oophorectomy, natural menopause, and breast cancer risk: an international prospective cohort of *BRCA1* and *BRCA2* mutation carriers

**DOI:** 10.1186/s13058-020-1247-4

**Published:** 2020-01-16

**Authors:** Nasim Mavaddat, Antonis C. Antoniou, Thea M. Mooij, Maartje J. Hooning, Bernadette A. Heemskerk-Gerritsen, GENEPSO, Catherine Noguès, Lilian Laborde, Emmanuel Breysse, Dominique Stoppa-Lyonnet, Marion Gauthier-Villars, Bruno Buecher, Olivier Caron, Emmanuelle Fourme-Mouret, Jean-Pierre Fricker, Christine Lasset, Valérie Bonadona, Pascaline Berthet, Laurence Faivre, Elisabeth Luporsi, Véronique Mari, Laurence Gladieff, Paul Gesta, Hagay Sobol, François Eisinger, Catherine Noguès, Michel Longy, Catherine Dugast, Chrystelle Colas, Isabelle Coupier, Pascal Pujol, Carole Corsini, Alain Lortholary, Philippe Vennin, Claude Adenis, Tan Dat Nguyen, Capucine Delnatte, Julie Tinat, Isabelle Tennevet, Jean-Marc Limacher, Christine Maugard, Yves-Jean Bignon, Liliane Demange, Clotilde Penet, Hélène Dreyfus, Odile Cohen-Haguenauer, Laurence Venat-Bouvet, Dominique Leroux, Hélène Dreyfus, Hélène Zattara-Cannoni, Sandra Fert-Ferrer, Odile Bera, Catherine Noguès, Marion Gauthier-Villars, Olivier Caron, Paul Gesta, Pascal Pujol, Alain Lortholary, EMBRACE, Steve Ellis, Daniel Barrowdale, Debra Frost, D. Gareth Evans, Louise Izatt, Julian Adlard, Ros Eeles, Carole Brewer, Marc Tischkowitz, Alex Henderson, Jackie Cook, Diana Eccles, HEBON, F. B. L. Hogervorst, J. M. Collée, C. J. van Asperen, A. R. Mensenkamp, M. G. E. M. Ausems, H. E. J. Meijers-Heijboer, K. van Engelen, M. J. Blok, J. C. Oosterwijk, J. Verloop, E. van den Broek, Klaartje van Engelen, Marian J. E. Mourits, Margreet G. E. M. Ausems, Linetta B. Koppert, John L. Hopper, Esther M. John, Wendy K. Chung, Irene L. Andrulis, Mary B. Daly, Saundra S. Buys, kConFab Investigators, Javier Benitez, Trinidad Caldes, Anna Jakubowska, Jacques Simard, Christian F. Singer, Yen Tan, Edith Olah, Marie Navratilova, Lenka Foretova, Anne-Marie Gerdes, Marie-José Roos-Blom, Flora E. Van Leeuwen, Brita Arver, Håkan Olsson, Rita K. Schmutzler, Christoph Engel, Karin Kast, Kelly-Anne Phillips, Mary Beth Terry, Roger L. Milne, David E. Goldgar, Matti A. Rookus, Nadine Andrieu, Douglas F. Easton, on behalf of IBCCS, kConFab, BCFR

**Affiliations:** 1 Centre for Cancer Genetic Epidemiology, Department of Public Health and Primary Care, Strangeways Research Laboratory, Worts Causeway, University of Cambridge, Cambridge, CBI 8RN UK; 2 Department of Epidemiology, Netherlands Cancer Institute, P.O. Box 90203, 1006 BE Amsterdam, The Netherlands; 3 Department of Medical Oncology, Family Center Clinic, Erasmus MC Cancer Institute, Rotterdam, The Netherlands; 4 DASC, Oncogénétique Clinique, Institut Paoli-Calmettes, Marseille, France; 5 Institut Curie, Service de Génétique, Paris, France; 6 Département de Médecine Oncologique, Gustave Roussy Hôpital Universitaire, Villejuif, France; 7 Centre Hospitalier, Service Régional d’Oncologie Génétique Poitou-Charentes, Niort, France; 8 Unité d’Oncogénétique, CHU Arnaud de Villeneuve, Montpellier, France; 9 Centre Catherine de Sienne, Service d’Oncologie Médicale, Nantes, France; 10 Genomic Medicine, Manchester Academic Health Sciences Centre, Division of Evolution and Genomic Sciences, Manchester University, Central Manchester, University Hospitals NHS Foundation Trust, Manchester, UK; 11 Clinical Genetics, Guy’s and St Thomas’ NHS Foundation Trust, London, UK; 12 Yorkshire Regional Genetics Service, Chapel Allerton Hospital and University of Leeds, Leeds, UK; 13 Oncogenetics Team, The Institute of Cancer Research and Royal Marsden NHS Foundation Trust, London, UK; 14 Department of Clinical Genetics, Royal Devon & Exeter Hospital, Exeter, UK; 15 Academic Department of Medical Genetics, National Institute for Health Research Cambridge Biomedical Research Centre, University of Cambridge, Cambridge, UK; 16 Institute of Genetic Medicine, Centre for Life, Newcastle Upon Tyne Hospitals NHS Trust, Newcastle upon Tyne, UK; 17 Sheffield Clinical Genetics Service, Sheffield Children’s Hospital, Sheffield, UK; 18 University of Southampton Faculty of Medicine, Southampton University Hospitals NHS Trust, Southampton, UK; 19 The Hereditary Breast and Ovarian Cancer Research Group Netherlands (HEBON), Coordinating Center: Netherlands Cancer Institute, Amsterdam, The Netherlands; 20 Department of Clinical Genetics, Amsterdam UMC, Vrije Universiteit Amsterdam, Amsterdam, Netherlands; 21 Department of Gynaecological Oncology, University Medical Center Groningen, University of Groningen, Groningen, The Netherlands; 22 Department of Genetics, University Medical Center Utrecht, Utrecht, The Netherlands; 23 Department of Surgical Oncology, Erasmus MC Cancer Institute, Rotterdam, The Netherlands; 24 Centre for Epidemiology and Biostatistics, Melbourne School of Population and Global Health, The University of Melbourne, Melbourne, VIC 3010 Australia; 25 Department of Medicine and Stanford Cancer Institute, Stanford University School of Medicine, Stanford, CA USA; 26 Departments of Pediatrics and Medicine, Columbia University Medical Center, New York, NY USA; 27 Herbert Irving Comprehensive Cancer Center, Columbia University Medical Center, New York, NY USA; 28 Department of Molecular Genetics, University of Toronto, Toronto, Ontario Canada; 29 Lunenfeld-Tanenbaum Research Institute, Sinai Health System, Toronto, Ontario Canada; 30 Department of Clinical Genetics, Fox Chase Cancer Center, Philadelphia, PA USA; 31 Department of Medicine, Huntsman Cancer Institute, University of Utah Health Sciences Center, Salt Lake City, UT USA; 32 Research Department, Peter MacCallum Cancer Centre, Melbourne, VIC Australia; 33 The Sir Peter MacCallum Department of Oncology, University of Melbourne, Parkville, Australia; 34 Human Genetics Group and Genotyping Unit, CEGEN, Human Cancer Genetics Programme, Spanish National Cancer Research Centre (CNIO), Madrid, Spain; 35 Molecular Oncology Laboratory, Hospital Clinico San Carlos, IdISSC, CIBERONC (ISCIII), Madrid, Spain; 36 Department of Genetics and Pathology, Pomeranian Medical University, Unii Lubelskiej 1, Szczecin, Poland; 37 Independent Laboratory of Molecular Biology and Genetic Diagnostics, Pomeranian Medical University, Unii Lubelskiej 1, Szczecin, Poland; 38 Genomics Center, Centre Hospitalier Universitaire de Québec, Université Laval Research Center, 2705 Laurier Boulevard, Quebec City, Quebec Canada; 39 Department of OB/GYN and Comprehensive Cancer Center, Medical University of Vienna, Waehringer Guertel 18-20, A 1090 Vienna, Austria; 40 Department of Molecular Genetics, National Institute of Oncology, Budapest, Hungary; 41 Department of Cancer Epidemiology and Genetics, Masaryk Memorial Cancer Institute, Zluty kopec 7, 65653 Brno, Czech Republic; 42 Department of Clinical Genetics, Rigshospitalet, Copenhagen University Hospital, Copenhagen, Denmark; 43 The Department of Oncology and Pathology, Karolinska Institute, 171 76 Stockholm, Sweden; 44 Department of Oncology, Lund University Hospital, Lund, Sweden; 45 Center for Familial Breast and Ovarian Cancer, Center for Integrated Oncology (CIO), Medical Faculty, University Hospital Cologne, Cologne, Germany; 46 Center for Molecular Medicine Cologne (CMMC), University of Cologne, Cologne, Germany; 47 Institute for Medical Informatics, Statistics and Epidemiology, University of Leipzig, Leipzig, Germany; 48 Department of Gynecology and Obstetrics, Medical Faculty and University Hospital Carl Gustav Carus, Technische Universität Dresden, Dresden, Germany; 49 National Center for Tumor Diseases (NCT), Partner Site Dresden, Dresden, Germany; 50 German Cancer Consortium (DKTK), Dresden and German Cancer Research Center (DKFZ), Heidelberg, Germany; 51 Department of Medical Oncology Peter MacCallum Cancer Centre, Locked Bag 1, A’Beckett St, East Melbourne, Victoria 8006 Australia; 52 Department of Epidemiology, Columbia University, New York, NY USA; 53 Cancer Epidemiology Division, Cancer Council Victoria, Melbourne, Victoria Australia; 54 Precision Medicine, School of Clinical Sciences at Monash Health, Monash University, Clayton, Victoria Australia; 55 Department of Dermatology, University of Utah School of Medicine, 30 North 1900 East, SOM 4B454, Salt Lake City, UT 841232 USA; 56 INSERM, U900, Paris, France; 57 Institut Curie, Paris, France; 58 Mines Paris Tech, Fontainebleau, France; 59 PSL Research University, Paris, France; 60 Centre for Cancer Genetic Epidemiology, Department of Oncology, Strangeways Research Laboratory, Worts Causeway, University of Cambridge, Cambridge, CBI 8RN UK

**Keywords:** Breast cancer, BRCA1, BRCA2, Mutation, Risk-reducing salpingo-oophorectomy

## Abstract

**Background:**

The effect of risk-reducing salpingo-oophorectomy (RRSO) on breast cancer risk for *BRCA1* and *BRCA2* mutation carriers is uncertain. Retrospective analyses have suggested a protective effect but may be substantially biased. Prospective studies have had limited power, particularly for *BRCA2* mutation carriers. Further, previous studies have not considered the effect of RRSO in the context of natural menopause.

**Methods:**

A multi-centre prospective cohort of 2272 *BRCA1* and 1605 *BRCA2* mutation carriers was followed for a mean of 5.4 and 4.9 years, respectively; 426 women developed incident breast cancer. RRSO was modelled as a time-dependent covariate in Cox regression, and its effect assessed in premenopausal and postmenopausal women.

**Results:**

There was no association between RRSO and breast cancer for *BRCA1* (HR = 1.23; 95% CI 0.94–1.61) or *BRCA2* (HR = 0.88; 95% CI 0.62–1.24) mutation carriers. For *BRCA2* mutation carriers, HRs were 0.68 (95% CI 0.40–1.15) and 1.07 (95% CI 0.69–1.64) for RRSO carried out before or after age 45 years, respectively. The HR for *BRCA2* mutation carriers decreased with increasing time since RRSO (HR = 0.51; 95% CI 0.26–0.99 for 5 years or longer after RRSO). Estimates for premenopausal women were similar.

**Conclusion:**

We found no evidence that RRSO reduces breast cancer risk for *BRCA1* mutation carriers. A potentially beneficial effect for *BRCA2* mutation carriers was observed, particularly after 5 years following RRSO. These results may inform counselling and management of carriers with respect to RRSO.

## Background

Women carrying germline mutations in *BRCA1* or *BRCA2* are at high risk of developing breast cancer and ovarian cancer [1, 2]. Mutation carriers undergo enhanced cancer surveillance and may be offered interventions including risk-reducing mastectomy (RRM) or risk-reducing salpingo-oophorectomy (RRSO). While RRSO substantially reduces the risk of developing ovarian cancer, its effect on breast cancer risk is uncertain. Some studies have reported substantial breast cancer risk reduction of up to 50% following RRSO [3–6]. However, these studies may have been subject to bias and confounding [7, 8]. Biases include ‘cancer-induced testing bias’, which can occur if mutation testing is conducted as a result of a breast cancer diagnosis and follow-up before DNA testing is included in the analysis, and ‘immortal person-time bias’, caused by excluding follow-up prior to RRSO uptake. Heemskerk-Gerritsen et al. found no evidence for an association between RRSO and breast cancer after eliminating several sources of bias [9, 10]. Prospective cohort studies can avoid such biases, but large studies with long follow-up are required to provide sufficient power.

Here, we report results from a large international collaborative, multi-centre, prospective cohort of 2272 *BRCA1* and 1605 *BRCA2* mutation carriers. We examined the association between RRSO and breast cancer risk according to the timing of RRSO relative to menopause and time since RRSO.

## Methods

### Study design and study population

We combined information from three consortia: The International BRCA1/2 Carrier Cohort Study (IBCCS), Kathleen Cuningham Foundation Consortium for Research Into Familial Breast Cancer (kConFab) Follow-Up Study, and Breast Cancer Family Registry (BCFR) (Tables 1 and 2, Additional file 1: Table S1) [11–15]. In total, 9856 *BRCA1/2* mutation carriers were included. Eighty-nine percent of participants were invited into the studies after receiving their clinical genetic test results, while 3% were recruited as an untested member of a mutation-carrying family and opted for a clinical test only after enrolment. Seven percent were tested in a research setting, and it was unknown whether or when they opted for a clinical test. Sixty-six percent of participants were enrolled through one of five ongoing nationwide studies in the UK and Ireland (Epidemiological Study of Familial Breast Cancer [EMBRACE]), France (Gene Etude Prospective Sein Ovaire [GENEPSO]), Netherlands (Hereditary Breast and Ovarian cancer study Netherlands [HEBON]), Australia and New Zealand (kConFab), and Austria (Medical University of Vienna [MUV]). Other studies were centre-based.

**Table 1:** Prospective cohort of *BRCA1* and *BRCA2* mutation carriers

IBCCS studies	*BRCA1* mutation carriers				*BRCA2* mutation carriers			
	Number of women	FUP time mean, years (sd)	BC, *N*	Mean age BC diagnosis, years	Number of women	FUP time mean, years (sd)	BC, *N*	Mean age BC diagnosis, years
EMBRACE	471	4.4 (3.0)	41	45.4	478	3.9 (2.5)	42	48.2
GENEPSO	486	3.6 (2.4)	46	45.8	325	3.2 (1.9)	18	48.8
HEBON	242	7.2 (3.6)	40	47.6	75	5.9 (2.8)	4	47.3
kConFab	325	6.7 (3.8)	55	42.2	288	6.4 (3.7)	38	50.5
BCFR	327	7.7 (4.4)	50	47.1	255	7.5 (4.3)	33	49.8
Other studies^a^	421	4.9 (3.2)	37	41.4	184	4.3 (2.9)	22	47.0
Total	2272	5.4 (3.7)	269	44.9	1605	4.9 (3.4)	157	49.0

*BC* breast cancer, *FUP* follow-up, *sd* standard deviation

^a^Other studies: MUV-Austria, INHERIT, OUH, GC-HBOC, NIO-Hungary, CNIO, HCSC, LUND-BRCA, STOCKHOLM-BRCA, IHCC, and MODSQUAD (see Additional file 1: Table S1 for details)

**Table 2:** Characteristics of the cohort of *BRCA1* and *BRCA2* mutation carriers

	*BRCA1* mutation carriers	*BRCA2* mutation carriers		
	Unaffected women (*N* = 2003)	Women with breast cancer (*N* = 269)	Unaffected women (*N* = 1448)	Women with breast cancer (*N* = 157)
Total person-years of follow-up	11,207	1134	7286	600
Person-years of follow-up (mean (sd))	5.60 (3.67)	4.21 (3.27)	5.03 (3.44)	3.82 (3.08)
Age at start of follow-up (mean (sd))	37.51 (11.80)	40.68 (10.25)	40.00 (12.53)	45.14 (10.11)
Age at diagnosis/censoring (mean (sd))	43.10 (12.28)	44.90 (10.33)	45.00 (13.00)	48.97 (10.30)
Reason for censoring				
Breast cancer	0	269	0	157
Ovarian cancer	49	3^a^	9	1^a^
Other cancer	45	5^a^	28	2^a^
RRM	299	–	181	–
Death	5	–	8	–
Unaffected at last follow-up time	1605	–	1222	–
Year of birth				
≤ 1960	604 (83.54)	119 (16.46)	500 (84.75)	90 (15.25)
> 1960	1399 (90.32)	150 (9.68)	948 (93.40)	67 (6.60)
Menopausal status				
Premenopausal at censoring^b^				
Last information^c^ after censoring	512	69	344	35
Last information before censoring^d^	585	58	473	35
Postmenopausal				
Natural menopause age known	194	27	182	31
Natural menopause age unknown	5	0	7	1
Post-hysterectomy	70	12	64	11
Unknown menopausal status	62	13	33	8
RRSO status at censoring				
No RRSO				
Last information after censoring	664	110	467	79
Last information before censoring	618	44	535	27
RRSO	721	115	446	51
As reason for menopause^e^	574	90	345	36
After natural menopause	101	18	76	10
After hysterectomy	46	7	25	5

*RRSO* risk-reducing salpingo-oophorectomy, *RRM* risk-reducing mastectomy

^a^Diagnosed at the same time as breast cancer

^b^Fifteen women did not report age at menopause but were older than 60 years at the end of follow-up

^c^Information from questionnaire and record linkage

^d^Age last known to be premenopausal mean 32.3 years, median 31 years for *BRCA1* mutation carriers: mean 33.9, median 34 years for *BRCA2* mutation carriers. Time between this age and end of censoring: mean 6.3, median 5 years for *BRCA1* and mean 6 years, median 5 years for *BRCA2* mutation carriers

^e^Seven women reported RRSO after age 60 years without first reporting natural menopause

### Study participants

Women were eligible if they were 18–80 years of age at recruitment and tested positive for a pathogenic *BRCA1* or *BRCA2* mutation, had no cancer history, and had retained both breasts at the date of genetic testing or study enrolment, whichever was last (*N* = 3886). One woman was excluded as she had been diagnosed with Turner syndrome and eight excluded as it was unclear whether they had had a hysterectomy or RRSO before recruitment.

### Data collection

Study participants were invited to complete a baseline questionnaire and a series of follow-up questionnaires. The questionnaires requested detailed information on known or suspected risk factors for breast and ovarian cancer, including family history, reproductive history, and surgical interventions including RRM or RRSO. The questionnaires also asked for information on age at last menstruation, whether the woman had had any period in the past year, the number of years/months since last menstruation, and reason(s) for the stopping of periods. Age at menopause for those who indicated no period in the past year was determined by adding 1 year to ‘age at last menstruation’. Women were considered premenopausal if they indicated that they had had a period in the past year, or if the ‘reason for periods stopping’ was medication, oral contraceptive use, pregnancy, or breast-feeding. Women reporting RRSO as the reason for menopause were considered premenopausal until RRSO. After hysterectomy, menopausal status was considered unknown.

In addition to questionnaires, some studies obtained RRSO information from medical records or linkage to a pathological registry. For the primary analysis, risk factor information was updated from all available sources, including post-diagnosis questionnaires and record linkage. Occurrence of breast cancer was derived from data from follow-up questionnaires and, for five studies, through linkage to cancer registries. Information on vital status was obtained from municipal or death registries, medical records, or family members.

Distributions of dates of breast cancer diagnosis and DNA testing are shown in Additional file 1: Table S2.

### Statistical analysis

We used Cox proportional hazards regression models to assess the association with risk of breast cancer. Follow-up started either at completion of baseline questionnaire or mutation testing, whichever was latest. The primary endpoint was breast cancer (invasive or in situ). Follow-up was censored at the earliest of RRM, diagnosis of breast cancer, ovarian cancer or any other cancer, treatment with chemotherapy or radiotherapy in the absence of information about cancer, reaching age 80 years, or death. For studies that used record linkage, follow-up was stopped at the date on which record linkage was conducted or considered complete. For GENEPSO, there was no linkage to cancer registries and women were censored at age at last questionnaire. Women diagnosed with breast cancer within 2 months of the start of follow-up were excluded from all analyses. RRM occurring within 1 year of breast cancer diagnosis were ignored. To investigate the association of RRSO with breast cancer risk in premenopausal women, women were also censored at natural menopause, hysterectomy, or reaching age 60 years. The association of RRSO with breast cancer risk after natural menopause was investigated by starting follow-up at the age of natural menopause. The association between age at natural menopause and breast cancer was investigated by also censoring at RRSO. For hormone replacement therapy (HRT) analyses, women were eligible if they had never used HRT before baseline and further censored at start of HRT.

A potential bias arises if completion of a subsequent questionnaire is related to RRSO uptake or cancer diagnosis. In order to address this possibility, sensitivity analyses were carried out in which RRSO status was changed at the date of the questionnaire in which the information on RRSO occurrence was reported, rather than the reported age at RRSO (except for the HEBON study, for which RRSO status was determined through record linkage). We also carried out sensitivity analysis excluding women with missing information on age or reason for menopause in the baseline questionnaire, even if this information was provided during follow-up (*n* = 514). Finally, we examined the effect of excluding women with prevalent RRSO at the start of follow-up (*n* = 403) (Additional file 1: Table S3).

Natural menopause and RRSO were coded as time-dependent covariates in a Cox regression model. In order to investigate the influence of age at RRSO on breast cancer risk, analyses were carried out separately for women experiencing RRSO before or after age 45 years. Analyses were also carried out estimating the hazard ratio for developing breast cancer for different time intervals following RRSO compared with no RRSO. The trend in HR by time since RRSO was evaluated by categorising the time following RRSO as < 2 years, 2–5 years, and > 5 years and fitting a time-varying parameter for this ordinal covariate (coded 0, 1, 2). We conducted separate analyses for *BRCA1* and *BRCA2* mutation carriers. We stratified for birth cohort and study (in six categories: EMBRACE, GENEPSO, HEBON, kConFab, BCFR, and other studies (Table 1)) and used robust variance estimation to account for familial clustering. We also assessed associations by birth cohort (1920–1960 or 1961–1992) and study and adjusted for potential confounders including family history of breast cancer in first- and second-degree relatives (collected either from the baseline questionnaire or from pedigrees provided by the genetics centres, and coded as unknown, none, one, or two or more breast cancers), family history of ovarian cancer (similarly defined), body mass index (BMI) at baseline (derived from self-reported height and weight), age at first birth (nulliparous, < 30 and ≥ 30), parity (nulliparous, 1, 2 or 3, and ≥ 4 full-term pregnancies), and HRT use (ever vs never, any formulation). The distribution of potential confounders in study subjects is shown in Additional file 1: Table S4. To test the heterogeneity between studies, fixed effect meta-analysis was carried out. Statistical analyses were performed using STATA v13 (StataCorp, College Station, TX). Statistical tests were considered significant based on two-sided hypothesis tests with *p* < 0.05.

## Results

### Cohort characteristics

Among 2272 *BRCA1* and 1605 *BRCA2* mutation carriers without a previous diagnosis of cancer or RRM, 269 *BRCA1* and 157 *BRCA2* mutation carriers were diagnosed with breast cancer during follow-up (mean follow-up time 5.4 and 4.9 years for *BRCA1* and *BRCA2*, respectively; Tables 1 and 2). In total, 836 (37%) *BRCA1* and 497 (31%) *BRCA2* mutation carriers reported RRSO, and 226 (10%) *BRCA1* and 221 (14%) *BRCA2* mutation carriers went through natural menopause, prior to censoring. Baseline demographics of the cohort are shown in Table 2 and Additional file 1: Table S4.

### Association between RRSO and breast cancer risk

**Fig. 1:**
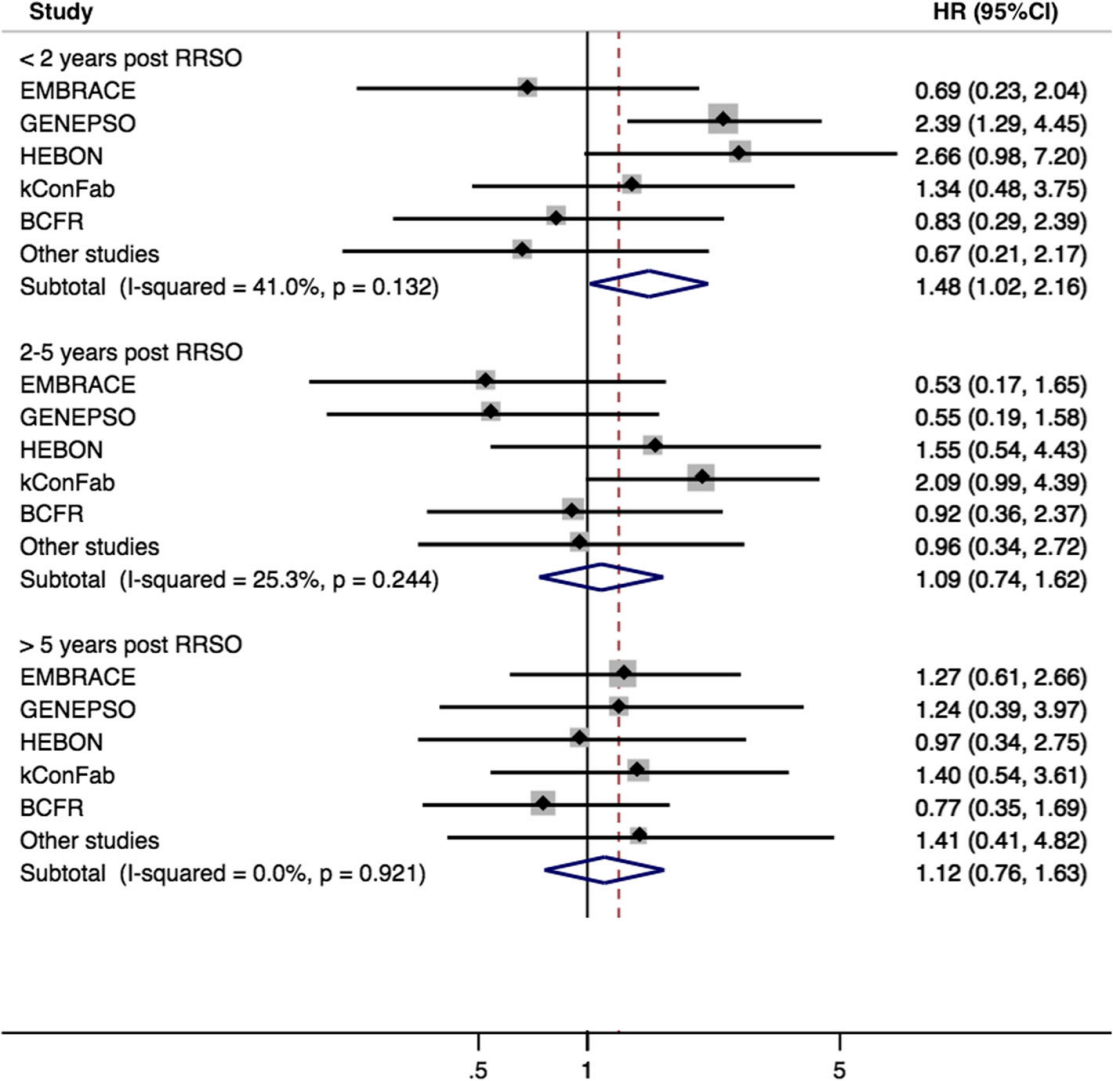
Association between risk-reducing salpingo-oophorectomy and breast cancer risk for *BRCA1* mutation carriers in each study centre category

**Fig. 2:**
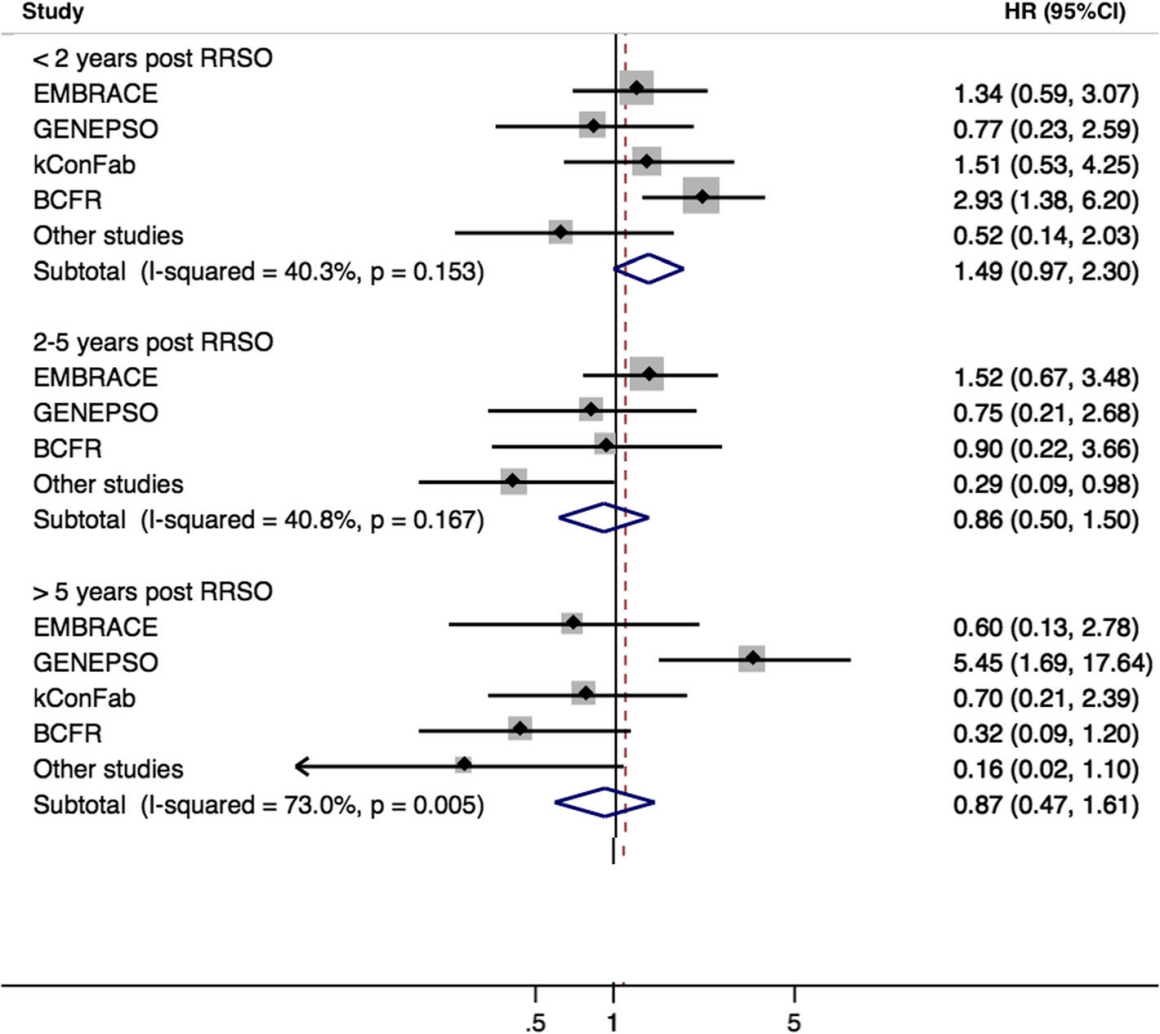
Association between risk-reducing salpingo-oophorectomy and breast cancer risk for *BRCA2* mutation carriers in each study centre category. HEBON and, for the 2–5-year category, kConFab were included in the “Other studies” category due to small numbers

In the primary analysis, the hazard ratio (HR) for the association between RRSO and breast cancer risk was 1.23 (95% CI 0.94–1.61) for *BRCA1* and 0.88 (95% CI 0.62–1.24) for *BRCA2* mutation carriers (Table 3). For *BRCA2* mutation carriers, the HR estimates were 0.68 (95% CI 0.40–1.15) and 1.07 (95% CI 0.69–1.64) for RRSO carried out before and after age 45 years, respectively. For *BRCA1* mutation carriers, the estimated HRs were close to 1 across varying times since RSSO (Table 3, Fig. 1), while for *BRCA2* mutation carriers, there was some evidence that the HR decreased with increasing time since RRSO (*p*-trend = 0.011) (Table 3). The HR estimates of greater than 1.0 less than 2 years after RRSO could reflect some inaccuracies in reporting the date of surgery. A protective association was observed for *BRCA2* mutation carriers 5 years after RRSO (HR = 0.51 (95% CI 0.26–0.99), *p* = 0.046, mean time between RRSO and end of follow-up, 9.5 years) (Table 3), although there were differences across studies (*p* value for heterogeneity = 0.005) (Fig. 2). The HR estimates were slightly lower for premenopausal *BRCA2* mutation carriers (Additional file 1: Table S5). There was no significant association between RRSO and breast cancer risk after natural menopause; however, only 221 *BRCA1* and 213 *BRCA2* mutation carriers were included in these analyses.

**Table 3:** Association between RRSO and breast cancer risk

	*BRCA1* mutation carriers				*BRCA2* mutation carriers			
	Person-years	BC	HR	95% CI	Person-years	BC	HR	95% CI
No RRSO	8353	154^a^	1.00	–	5769	106^b^	1.00	–
RRSO at any age (years)	3988	115^a^	1.23	0.94–1.61	2117	51^b^	0.88	0.62–1.24
≤ 45	2205	64	1.19	0.88–1.61	964	17	0.68	0.40–1.15
> 45	1783	51	1.34	0.89–2.02	1153	34	1.07	0.69–1.64
Time since RRSO (years)								
< 2	1111	40	1.43	1.01–2.03	694	24	1.29	0.82–2.02
2–5	1261	32	1.06	0.71–1.57	722	17	0.82	0.48–1.38
> 5	1616	43	1.18	0.81–1.71	701	10	0.51	0.26–0.99

A Cox regression model was used adjusting for country, stratified by year of birth (≤ 1960, ≥ 1961) and with robust standard errors (clustering by family)

*BC* breast cancer, *RRSO* risk-reducing salpingo-oophorectomy, *HR* hazard ratio

^a^Among *BRCA1* mutation carriers, tumour pathology was unknown for 5 women without RRSO and 9 following RRSO

^b^Among *BRCA2* mutation carriers, tumour pathology was unknown for 12 women without RRSO and 7 following RRSO

The results of the sensitivity analyses were broadly similar to the main analyses (Additional file 1: Tables S6-S8).

Analyses were also adjusted for potential confounders: parity, BMI, age at first birth, and family history of breast or ovarian cancer. Association between breast cancer risk factors and uptake of RRSO are shown in Additional file 1: Tables S9 and S10. In the analyses adjusted for these covariates, the estimated effect sizes were similar to those in the unadjusted analyses (Additional file 1: Table S11). Effect estimates for the analyses carried out among women who had never taken HRT were similar to those in the primary analyses (Additional file 1: Tables S12 and S13).

## Discussion

Reliable estimation of the association between uptake and timing of RRSO and breast cancer risk is critical for informing counselling and clinical management of *BRCA1* and *BRCA2* mutation carriers. Our study of 3877 mutation carriers with 426 incident breast cancer cases is the largest prospective cohort to date and the first prospective study investigating breast cancer risk after RRSO for *BRCA1* and *BRCA2* mutation carriers in the context of menopausal status.

We found no significant association between RRSO and breast cancer risk for *BRCA1* or *BRCA2* mutation carriers, although the point estimate for the association for *BRCA2* mutation carriers was less than 1 (HR = 0.88 (95% CI 0.62–1.24)) and lower when RRSO was carried out before the age of 45 (HR = 0.68 (95% CI 0.40–1.15) vs 1.07 (95% CI 0.69–1.64) after age 45). Our overall results are inconsistent with previous reports of ~ 50% reduction in breast cancer risk for *BRCA1* mutation carriers [3, 6] but more consistent with a study by Kotsopolous et al. reporting risk reduction only for younger *BRCA2* mutation carriers [16]. The latter study was prospective, but its results were based on only 3 breast cancers in women aged under 50 years; our study included more than twice as many *BRCA2* mutation carriers overall, and the analyses were based on 31 incident breast cancers in premenopausal *BRCA2* mutation carriers. In addition, we investigated associations by time since RRSO. For *BRCA2* mutation carriers, we observed a decreasing trend in HR with increasing time since RRSO; relative to women who did not have an RSSO, the estimated HR > 5 years following RSSO was 0.51. In contrast, for *BRCA1* mutation carriers, the HR was close to 1 at all times since RRSO.

While this is the largest prospective cohort of mutation carriers to date, the number of breast cancer cases was still limited, and hence, the confidence limits for the HR estimates were wide. Additional data would be needed to determine whether or not there is a modest protective effect of RRSO for *BRCA1* mutation carriers and whether the suggested protective effect in *BRCA2* mutation carriers is real.

There was some suggestion of differences in estimated effect size among studies for *BRCA1* mutation carriers in the < 2-year and ‘2–5-year’ post-RRSO groups (Fig. 1), but the heterogeneity was not statistically significant. For *BRCA2* mutation carriers, there was statistically significant heterogeneity in the RRSO > 5 years group (Fig. 2); this appeared to be driven by a large effect size in GENEPSO, based on only two breast cancers. Studies differed in methodology (including frequency of questionnaires, assessment of breast cancers or RRSO, loss to follow-up, and mean follow-up time). EMBRACE, GENEPSO, and HEBON ascertained participants through cancer genetics clinics, while BCFR used both clinic- and population-based recruitment. There was also some geographical variation in the uptake and age at RRSO (Additional file 1: Table S3). However, the cohorts were recruited and followed up over broadly similar periods (Additional file 1: Table S2).

The strength of this study is its prospective design. Many of the biases identified in previous reports were addressed [7, 9, 17, 18]. We avoided cancer testing-induced bias by starting follow-up after mutation testing. Women were not selected for inclusion in the study on the basis of RRSO status, and time-dependent covariates were used to examine the effect of RRSO on breast cancer risk. While it is impossible to rule out bias due to unmeasured confounders in an observational study, adjustment for potential confounders (family history of breast and ovarian cancer, parity, age at first birth, and BMI) did not materially influence the results.

In the general population, HRT use is associated with an increased risk of breast cancer. HRT use after RRSO may therefore attenuate the risk reduction due to RRSO. Our preliminary analyses restricted to the subset of women not reporting HRT use gave broadly similar results (Additional file 1: Table S13), but the effects of HRT post-RRSO will need to be further investigated in larger cohorts and studies that consider the type, formulation, and duration of HRT use.

While often considered the ‘gold standard’ for investigating exposure-disease associations, prospective cohort studies are still prone to biases resulting from missing data, loss to follow-up, and informative censoring. In particular, there are gaps in data collection between questionnaires and between the last questionnaire and censoring, during which risk factors can change. We carried out sensitivity analyses in which risk factors were scored according to the most recent questionnaire, thus treating equally women who reached a particular questionnaire follow-up and those who dropped out before reaching this time point. This analysis avoids differential scoring of risk factors between those who developed breast cancer and those who did not develop breast cancer but would be expected to result in loss of power. We also carried out sensitivity analyses excluding two studies, kConFab and BCFR, as these studies were included in a recent analysis of RRSO in women with a family history of breast cancer (Additional file 1: Table S14) [19]. The results of these analyses were almost identical to those from the primary analyses. Reporting of natural menopause is also subject to recall bias and measurement error, and for about half of women reporting premenopausal status, the questionnaires did not cover the entire follow-up period.

A potential bias in the estimate of the RRSO association could arise if the timing of uptake of RRSO was related to the imminent transition to menopause. If there was a protective effect of early natural menopause on cancer risk for mutation carriers, this could result in an overestimation of the RRSO effect in the overall analysis. However, we found no evidence for a strong association between age at natural menopause and breast cancer risk (Additional file 1: Table S15), so any such bias is likely to be small.

Recent genome-wide association analyses have shown that age at natural menopause is partially determined by variants in DNA repair genes, including common coding variants in *BRCA1* [20]. Some studies have suggested that natural menopause occurs at a younger age for *BRCA1* and *BRCA2* mutation carriers compared with women from the general population [21–24] and that *BRCA1* mutation carriers have reduced ovarian reserve, and consequently a shortened reproductive lifespan, compared with non-carriers [25]. *BRCA1* mutation carriers have also been found to be more likely to have occult ovarian insufficiency [21]. The effect of menopause on breast cancer risk might therefore differ in mutation carriers compared with the general population.

It is plausible that oophorectomy may reduce breast cancer risk in *BRCA2* mutation carriers but not in *BRCA1* mutation carriers. Breast cancer incidence peaks or plateaus at a younger age (early 40s) in *BRCA1* than *BRCA2* mutation carriers [2], perhaps suggesting that much of the carcinogenic process in *BRCA1* mutation carriers takes place before women typically have RRSO and could influence disease incidence. In addition, *BRCA2*-related tumours are mainly oestrogen receptor (ER)-positive, and *BRCA1*-related tumours are mainly ER-negative. Previous analyses have suggested that in the general population, the association of early menopause with reduced breast cancer risk is larger for ER-positive disease [26]. Future analyses stratified by molecular subtype of breast cancer should help delineate mechanisms underlying this difference.

Optimum timing of RRSO should take into account reported age-specific incidences of ovarian cancer among *BRCA1* and *BRCA2* mutation carriers [2]. National Comprehensive Cancer Network (NCCN) guidelines for example recommend RRSO for *BRCA1* mutation carriers, typically between 35 and 40 years of age and upon completion of child-bearing; for *BRCA2* mutation carriers, these guidelines suggest that it is reasonable to delay RRSO until age 40–45 years [27]. Cancer Australia clinical guidelines recommend RRSO in confirmed mutation carriers around age 40 years, while considering individual risk and circumstances [28]. Adverse effects of RRSO at a young age, including reduced quality of life, cardiovascular disease, and osteoporosis, should also be taken into consideration. The results of our study indicate that caution should be exercised in conveying information on the risk of breast cancer after RRSO, and emphasise the need for continued surveillance for breast cancer following RRSO for women who do not opt for risk-reducing mastectomy,

The results of our analyses further suggest that continued follow-up of prospective cohorts of mutation carriers, with linkage to end-point and risk factor data, are required. These findings need replication in larger studies of *BRCA1* and *BRCA2* mutation carriers, particularly including more women in whom RRSO was carried out at a young age. More complete data on factors such as a family history of breast or ovarian cancer would be valuable. Prospective studies with long-term follow-up will also be important for analysing the association between HRT use and breast cancer risk following RRSO, as limited data have been available to date. In addition, RRSO has been reported to reduce mortality from breast cancer [29–31], and there is some evidence that breast cancers arising after RRSO are more indolent than those arising without RRSO [32]. Prospective studies of survival after RRSO would further inform counselling and management of *BRCA1* and *BRCA2* mutation carriers.

## Conclusions

While the primary purpose of RRSO is the prevention of ovarian cancer, information on the effect of RRSO on breast cancer risk is essential for clinical decision-making, including the decision to undergo a risk-reducing mastectomy. Our results suggest that a protective effect of RRSO for *BRCA2* mutation carriers may manifest five or more years after surgery. While we cannot rule out an effect of RRSO on breast cancer risk for *BRCA1* mutation carriers, this effect is unlikely to be as large.

## Supplementary information

**Additional file 1 :Table S1.** Studies and samples included in the prospective cohort of *BRCA1* and *BRCA2* mutation carriers. **Table S2.** Distributions of dates of breast cancer diagnosis, DNA test and start of follow-up in the prospective cohort.** Table S3.** Characteristics of reported Risk-Reducing Salpingo-oophorectomy. **Table S4.** Characteristics of cohort of *BRCA1* and *BRCA2* mutation carriers. **Table S5.** Association between RRSO and breast cancer by menopausal status. **Table S6.** Association between RRSO and breast cancer (sensitivity analysis with RRSO status changing at the age at the questionnaire with information on RRSO status changes (all studies except HEBON)). **Table S7.** Association between RRSO and breast cancer (sensitivity analysis dropping individuals with missing information at baseline). **Table S8.** Association between RRSO and breast cancer among *BRCA1* and *BRCA2* mutation carriers (sensitivity analysis excluding women with RRSO before baseline). **Table S9.** Association between family history of breast cancer and family history of ovarian cancer and RRSO uptake. **Table S10.** Association between parity, age at first birth, and body mass index and RRSO uptake. **Table S11.** Association between RRSO and breast cancer adjusting for Body Mass Index, family history of breast cancer, family history of ovarian cancer, parity and age at first birth. **Table S12.** Hormone replacement therapy use among women in the cohort. **Table S13.** Association between RRSO and breast cancer among women not exposed to hormone replacement therapy. **Table S14.** Association between RRSO and breast cancer (excluding kConFab/BCFR). **Table S15.** Association between natural menopause and breast cancer (censoring at RRSO). Ethics Committee Approvals

## Data Availability

The dataset supporting the conclusions of this article are available upon reasonable request. Requests should be made to Dr. M Rookus (NKI, Amsterdam, NL; m.rookus@nki.nl).

## Abbreviations

BMI: Body mass index
EMBRACE: Epidemiological Study of Familial Breast Cancer
GENEPSO: Gene Etude Prospective Sein Ovaire
HEBON: Hereditary Breast and Ovarian cancer study Netherlands
HRT: Hormone replacement therapy
IBCCS: International BRCA1/2 Carrier Cohort Study
kConFab: Kathleen Cuningham Foundation Consortium for Research Into Familial Breast Cancer
RRM: Risk-reducing mastectomy
RRSO: Risk-reducing salpingo-oophorectomy
