# Correction to: Risk-reducing salpingo-oophorectomy, natural menopause, and breast cancer risk: an international prospective cohort of BRCA1 and BRCA2 mutation carriers

**DOI:** 10.1186/s13058-020-01259-w

**Published:** 2020-02-26

**Authors:** Nasim Mavaddat, Antonis C. Antoniou, Thea M. Mooij, Maartje J. Hooning, Bernadette A. Heemskerk-Gerritsen, GENEPSO, Catherine Noguès, Marion Gauthier-Villars, Olivier Caron, Paul Gesta, Pascal Pujol, Alain Lortholary, EMBRACE, Daniel Barrowdale, Debra Frost, D. Gareth Evans, Louise Izatt, Julian Adlard, Ros Eeles, Carole Brewer, Marc Tischkowitz, Alex Henderson, Jackie Cook, Diana Eccles, HEBON, Klaartje van Engelen, Marian J. E. Mourits, Margreet G. E. M. Ausems, Linetta B. Koppert, John L. Hopper, Esther M. John, Wendy K. Chung, Irene L. Andrulis, Mary B. Daly, Saundra S. Buys, kConFab Investigators, Javier Benitez, Trinidad Caldes, Anna Jakubowska, Jacques Simard, Christian F. Singer, Yen Tan, Edith Olah, Marie Navratilova, Lenka Foretova, Anne-Marie Gerdes, Marie-José Roos-Blom, Flora E. Van Leeuwen, Brita Arver, Håkan Olsson, Rita K. Schmutzler, Christoph Engel, Karin Kast, Kelly-Anne Phillips, Mary Beth Terry, Roger L. Milne, David E. Goldgar, Matti A. Rookus, Nadine Andrieu, Douglas F. Easton, on behalf of IBCCS, kConFab and BCFR

**Affiliations:** 1 Centre for Cancer Genetic Epidemiology, Department of Public Health and Primary Care, Strangeways Research Laboratory, Worts Causeway, University of Cambridge, Cambridge, CBI 8RN UK; 2 Department of Epidemiology, Netherlands Cancer Institute, P.O. Box 90203, 1006 BE Amsterdam, The Netherlands; 3 Department of Medical Oncology, Family Center Clinic, Erasmus MC Cancer Institute, Rotterdam, The Netherlands; 4 DASC, Oncogénétique Clinique, Institut Paoli-Calmettes, Marseille, France; 5 Institut Curie, Service de Génétique, Paris, France; 6 Département de Médecine Oncologique, Gustave Roussy Hôpital Universitaire, Villejuif, France; 7 Centre Hospitalier, Service Régional d’Oncologie Génétique Poitou-Charentes, Niort, France; 8 Unité d’Oncogénétique, CHU Arnaud de Villeneuve, Montpellier, France; 9 Centre Catherine de Sienne, Service d’Oncologie Médicale, Nantes, France; 10 Genomic Medicine, Manchester Academic Health Sciences Centre, Division of Evolution and Genomic Sciences, Manchester University, Central Manchester, University Hospitals NHS Foundation Trust, Manchester, UK; 11 Clinical Genetics, Guy’s and St Thomas’ NHS Foundation Trust, London, UK; 12 Yorkshire Regional Genetics Service, Chapel Allerton Hospital and University of Leeds, Leeds, UK; 13 Oncogenetics Team, The Institute of Cancer Research and Royal Marsden NHS Foundation Trust, London, UK; 14 Department of Clinical Genetics, Royal Devon & Exeter Hospital, Exeter, UK; 15 Academic Department of Medical Genetics, National Institute for Health Research Cambridge Biomedical Research Centre, University of Cambridge, Cambridge, UK; 16 Institute of Genetic Medicine, Centre for Life, Newcastle Upon Tyne Hospitals NHS Trust, Newcastle upon Tyne, UK; 17 Sheffield Clinical Genetics Service, Sheffield Children’s Hospital, Sheffield, UK; 18 University of Southampton Faculty of Medicine, Southampton University Hospitals NHS Trust, Southampton, UK; 19 The Hereditary Breast and Ovarian Cancer Research Group Netherlands (HEBON), Coordinating Center: Netherlands Cancer Institute, Amsterdam, The Netherlands; 20 Department of Clinical Genetics, Amsterdam UMC, Vrije Universiteit Amsterdam, Amsterdam, Netherlands; 21 Department of Gynaecological Oncology, University Medical Center Groningen, University of Groningen, Groningen, The Netherlands; 22 Department of Genetics, University Medical Center Utrecht, Utrecht, The Netherlands; 23 Department of Surgical Oncology, Erasmus MC Cancer Institute, Rotterdam, The Netherlands; 24 Centre for Epidemiology and Biostatistics, Melbourne School of Population and Global Health, The University of Melbourne, Melbourne, VIC 3010 Australia; 25 Department of Medicine and Stanford Cancer Institute, Stanford University School of Medicine, Stanford, CA USA; 26 Departments of Pediatrics and Medicine, Columbia University Medical Center, New York, NY USA; 27 Herbert Irving Comprehensive Cancer Center, Columbia University Medical Center, New York, NY USA; 28 Department of Molecular Genetics, University of Toronto, Toronto, Ontario Canada; 29 Lunenfeld-Tanenbaum Research Institute, Sinai Health System, Toronto, Ontario Canada; 30 Department of Clinical Genetics, Fox Chase Cancer Center, Philadelphia, PA USA; 31 Department of Medicine, Huntsman Cancer Institute, University of Utah Health Sciences Center, Salt Lake City, UT USA; 32 Research Department, Peter MacCallum Cancer Centre, Melbourne, VIC Australia; 33 The Sir Peter MacCallum Department of Oncology, University of Melbourne, Parkville, Australia; 34 Human Genetics Group and Genotyping Unit, CEGEN, Human Cancer Genetics Programme, Spanish National Cancer Research Centre (CNIO), Madrid, Spain; 35 Molecular Oncology Laboratory, Hospital Clinico San Carlos, IdISSC, CIBERONC (ISCIII), Madrid, Spain; 36 Department of Genetics and Pathology, Pomeranian Medical University, Unii Lubelskiej 1, Szczecin, Poland; 37 Independent Laboratory of Molecular Biology and Genetic Diagnostics, Pomeranian Medical University, Unii Lubelskiej 1, Szczecin, Poland; 38 Genomics Center, Centre Hospitalier Universitaire de Québec, Université Laval Research Center, 2705 Laurier Boulevard, Quebec City, Quebec Canada; 39 Department of OB/GYN and Comprehensive Cancer Center, Medical University of Vienna, Waehringer Guertel 18-20, A 1090 Vienna, Austria; 40 Department of Molecular Genetics, National Institute of Oncology, Budapest, Hungary; 41 Department of Cancer Epidemiology and Genetics, Masaryk Memorial Cancer Institute, Zluty kopec 7, 65653 Brno, Czech Republic; 42 Department of Clinical Genetics, Rigshospitalet, Copenhagen University Hospital, Copenhagen, Denmark; 43 The Department of Oncology and Pathology, Karolinska Institute, 171 76 Stockholm, Sweden; 44 Department of Oncology, Lund University Hospital, Lund, Sweden; 45 Center for Familial Breast and Ovarian Cancer, Center for Integrated Oncology (CIO), Medical Faculty, University Hospital Cologne, Cologne, Germany; 46 Center for Molecular Medicine Cologne (CMMC), University of Cologne, Cologne, Germany; 47 Institute for Medical Informatics, Statistics and Epidemiology, University of Leipzig, Leipzig, Germany; 48 Department of Gynecology and Obstetrics, Medical Faculty and University Hospital Carl Gustav Carus, Technische Universität Dresden, Dresden, Germany; 49 National Center for Tumor Diseases (NCT), Partner Site Dresden, Dresden, Germany; 50 German Cancer Consortium (DKTK), Dresden and German Cancer Research Center (DKFZ), Heidelberg, Germany; 51 Department of Medical Oncology Peter MacCallum Cancer Centre, Locked Bag 1, A’Beckett St, East Melbourne, Victoria 8006 Australia; 52 Department of Epidemiology, Columbia University, New York, NY USA; 53 Cancer Epidemiology Division, Cancer Council Victoria, Melbourne, Victoria Australia; 54 Precision Medicine, School of Clinical Sciences at Monash Health, Monash University, Clayton, Victoria Australia; 55 Department of Dermatology, University of Utah School of Medicine, 30 North 1900 East, SOM 4B454, Salt Lake City, UT 841232 USA; 56 INSERM, U900, Paris, France; 57 Institut Curie, Paris, France; 58 Mines Paris Tech, Fontainebleau, France; 59 PSL Research University, Paris, France; 60 Centre for Cancer Genetic Epidemiology, Department of Oncology, Strangeways Research Laboratory, Worts Causeway, University of Cambridge, Cambridge, CBI 8RN UK

**Correction to: Breast Cancer Res**

**https://doi.org/10.1186/s13058-020-1247-4**

After publication of the original article [1], we were notified that columns in Table 2 were erroneously displayed.

The correction version of Table 2 is shown below.

**Table 2:** Characteristics of cohort of *BRCA1* and *BRCA2* mutation carriers

	*BRCA1* mutation carriers		*BRCA2* mutation carriers	
	Unaffected women (*N* = 2003)	Women with breast cancer (*N* = 269)	Unaffected women (*N* = 1448)	Women with breast cancer (*N* = 157)
Total Person-years of follow-up	11,207	1134	7286	600
Person-years of follow-up (mean (sd))	5.60 (3.67)	4.21 (3.27)	5.03 (3.44)	3.82 (3.08)
Age at start of follow-up (mean (sd))	37.51 (11.80)	40.68 (10.25)	40.00 (12.53)	45.14 (10.11)
Age at diagnosis/censoring (mean (sd))	43.10 (12.28)	44.90 (10.33)	45.00 (13.00)	48.97 (10.30)
Reason for censoring				
Breast cancer	0	269	0	157
Ovarian cancer	49	3^*a*^	9	1^a^
Other cancer	45	5^*a*^	28	2^a^
RRM	299	–	181	–
Death	5	–	8	–
Unaffected at last follow-up time	1605	–	1222	–
Year of birth				
< =1960	604 (83.54)	119 (16.46)	500 (84.75)	90 (15.25)
> 1960	1399 (90.32)	150 (9.68)	948 (93.40)	67 (6.60)
Menopausal status				
Premenopausal at censoring^*b*^				
Last information^c^ after censoring	512	69	344	35
Last information before censoring^*d*^	585	58	473	35
Postmenopausal				
Natural menopause age known	194	27	182	31
Natural menopause age unknown	5	0	7	1
Post hysterectomy	70	12	64	11
Unknown menopausal status	62	13	33	8
RRSO status at censoring				
No RRSO				
Last information after censoring	664	110	467	79
Last information before censoring	618	44	535	27
RRSO	721	115	446	51
As reason for menopause^*e*^	574	90	345	36
After natural menopause	101	18	76	10
After hysterectomy	46	7	25	5

*Abbreviations*: *RRSO* Risk-Reducing Salpingo-oophorectomy, *RRM* Risk Reducing Mastectomy

^*a*^Diagnosed at the same time as breast cancer

^*b*^Fifteen women did not report age at menopause but were older than 60 years at the end of follow-up

^*c*^Information from questionnaire and record linkage

^*d*^Age last known to be premenopausal: mean 32.3 years, median 31 years for *BRCA1* mutation carriers: mean 33.9, median 34 years for *BRCA2* mutation carriers. Time between this age and end of censoring: mean 6.3, median 5 years for *BRCA1* and mean 6 years, median 5 years for *BRCA2* mutation carriers

^*e*^Seven women reported RRSO after age 60 years without first reporting natural menopause
