# Supplementary material for: Risk-reducing salpingo-oophorectomy, natural menopause, and breast cancer risk: an international prospective cohort of BRCA1 and BRCA2 mutation carriers
Source: Breast Cancer Res. 2020 Jan 16;22:8. doi: 10.1186/s13058-020-1247-4 (PMC6966793; doi:10.1186/s13058-020-1247-4)
Supplement: Supplementary file 1 — Additional file 1 :Table S1. Studies and samples included in the prospective cohort of BRCA1 and BRCA2 mutation carriers. Table S2. Distributions of dates of breast cancer diagnosis, DNA test and start of follow-up in the prospective cohort. Table S3. Characteristics of reported Risk-Reducing Salpingo-oophorectomy. Table S4. Characteristics of cohort of BRCA1 and BRCA2 mutation carriers. Table S5. Association between RRSO and breast cancer by menopausal status. Table S6. Association between RRSO and breast cancer (sensitivity analysis with RRSO status changing at the age at the questionnaire with information on RRSO status changes (all studies except HEBON)). Table S7. Association between RRSO and breast cancer (sensitivity analysis dropping individuals with missing information at baseline). Table S8. Association between RRSO and breast cancer among BRCA1 and BRCA2 mutation carriers (sensitivity analysis excluding women with RRSO before baseline). Table S9. Association between family history of breast cancer and family history of ovarian cancer and RRSO uptake. Table S10. Association between parity, age at first birth, and body mass index and RRSO uptake. Table S11. Association between RRSO and breast cancer adjusting for Body Mass Index, family history of breast cancer, family history of ovarian cancer, parity and age at first birth. Table S12. Hormone replacement therapy use among women in the cohort. Table S13. Association between RRSO and breast cancer among women not exposed to hormone replacement therapy. Table S14. Association between RRSO and breast cancer (excluding kConFab/BCFR). Table S15. Association between natural menopause and breast cancer (censoring at RRSO). Ethics Committee Approvals [file 13058_2020_1247_MOESM1_ESM.docx]

**Supplementary Tables**

**Table S1. Studies and samples included in the prospective cohort of *BRCA1* and *BRCA2* mutation carriers**

| **STUDY** | ***BRCA1* mutation carriers** | ***BRCA2* mutation carriers** | **Total** |
| --- | --- | --- | --- |
| EMBRACE | 471 | 478 | 949 |
| GENEPSO | 486 | 325 | 811 |
| HEBON | 242 | 75 | 317 |
| kConFab | 325 | 288 | 613 |
| BCFR | 327 | 255 | 582 |
| MUV-Austria | 65 | 19 | 84 |
| INHERIT | 30 | 31 | 61 |
| OUH | 47 | 27 | 74 |
| GC-HBOC | 35 | 19 | 54 |
| NIO-Hungary | 25 | 12 | 37 |
| CNIO | 7 | 6 | 13 |
| HCSC | 31 | 23 | 54 |
| LUND-BRCA | 58 | 17 | 75 |
| STOCKHOLM-BRCA | 13 | 2 | 15 |
| IHCC | 27 | 0 | 27 |
| MODSQUAD | 83 | 28 | 111 |
| Total | 2272 | 1605 | 3877 |

**Table S2 – Distributions of dates of breast cancer diagnosis, DNA test and start of follow-up in the prospective cohort**

| **Study** | **Dates of breast**  **cancer diagnosis** | **Dates of**  **DNA test** |  | **Dates of start of follow-up** |
| --- | --- | --- | --- | --- |
|  |  |  |  |  |
|  | Min - Max | Min - Max | N* | Min - Max |
| ***BRCA1* mutation carriers** | |  |  |  |
| EMBRACE | 2000 - 2012 | 1995 - 2011 | 471 | 1997 - 2011 |
| GENEPSO | 2001 - 2011 | 1994 - 2009 | 486 | 2000 – 2010 |
| HEBON | 2002 - 2012 | 1995 - 2007 | 242 | 1996 – 2009 |
| kConFab | 1999 - 2011 | 1996 - 2011 | 240 | 1997 – 2010 |
| BCFR | 1995 - 2013 | ---- | 0 | 1993 – 2010 |
| Other studies | 1997 - 2012 | 1994 - 2011 | 421 | 1994 – 2011 |
| Total | 1995 - 2013 | 1994 - 2011 | 1860 | 1993 – 2011 |
| ***BRCA2* mutation carriers** | |  |  |  |
| EMBRACE | 2003 - 2012 | 1996 - 2011 | 478 | 1999 - 2011 |
| GENEPSO | 2002 - 2009 | 1997 - 2010 | 325 | 2000 - 2010 |
| HEBON | 2002 - 2010 | 1996 - 2008 | 75 | 2000 - 2009 |
| kConFab | 1998 - 2012 | 1998 - 2011 | 193 | 1997 - 2011 |
| BCFR | 1996 - 2013 | ---- | 0 | 1993 - 2010 |
| Other studies | 2001 - 2012 | 1996 - 2011 | 184 | 1996 - 2011 |
| Total | 1996 - 2013 | 1996 - 2011 | 1255 | 1993 - 2011 |

*N = number of individuals with DNA testing date available

**Table S3. Characteristics of reported Risk-Reducing Salpingo-oophorectomy**

|  |  | **All RRSO before censoring** | | | **RRSO before start of follow-up** | | | **RRSO during follow-up** | | |
| --- | --- | --- | --- | --- | --- | --- | --- | --- | --- | --- |
| Study | Without RRSO | Number of  RRSO (%)^a^ | Mean age RRSO (years) | Mean time between RRSO and BC (years) | Number  of  RRSO (%)^b^ | Mean age RRSO (years) | Mean time between RRSO and BC (years) | Number  of  RRSO | Mean age RRSO (years) | Mean time between RRSO and BC (years) |
| ***BRCA1* mutation carriers** | | | | | | | | | | |
| EMBRACE | 295 | 176(37%) | 43.9 | 7.4 | 62­­ (35%) | 43.8 | 9.4 | 114 | 43.9 | 5.3 |
| GENEPSO | 306 | 180(37%) | 46.1 | 3.1 | 31 (17%) | 47.3 | 7.2 | 149 | 45.9 | 2.0 |
| HEBON | 109 | 133(55%) | 45.8 | 5.7 | 85 (64%) | 48.6 | 6.9 | 48 | 41.0 | 4.1 |
| kConFab | 231 | 94 (29%) | 43.8 | 8.0 | 19 (20%) | 39.6 | 15.6 | 75 | 44.9 | 4.5 |
| BCFR | 207 | 120(37%) | 44.2 | 8.7 | 35 (29%) | 43.8 | 14.6 | 85 | 44.3 | 5.3 |
| Other studies | 288 | 133(32%) | 44.7 | 6.2 | 38 (29%) | 46.1 | 10.6 | 95 | 44.2 | 4.3 |
| Total | 1436 | 836(37%) | 44.8 | 6.3 | 270(32%) | 45.7 | 10.1 | 566 | 44.4 | 4.0 |
| ***BRCA2* mutation carriers** | | | | | | | | | | |
| EMBRACE | 303 | 175(37%) | 46.8 | 3.4 | 45 (26%) | 46.6 | 4.8 | 130 | 46.8 | 2.9 |
| GENEPSO | 243 | 82 (25%) | 49.3 | 6.9 | 7 (9%) | 41.4 | 19.0 | 75 | 50.1 | 2.1 |
| HEBON | 34 | 41 (55%) | 48.9 | 3.0 | 22 (54%) | 51.2 | 3.0 | 19 | 46.1 | - |
| kConFab | 225 | 63 (22%) | 47.1 | 8.5 | 19 (30%) | 43.4 | 16.5 | 44 | 48.7 | 2.5 |
| BCFR | 168 | 87 (37%) | 45.5 | 3.8 | 19 (22%) | 44.2 | 11.0 | 68 | 45.8 | 2.3 |
| Other studies | 135 | 49 (27%) | 47.4 | 3.8 | 21 (43%) | 48.5 | 4.0 | 28 | 46.6 | 2.0 |
| Total | 1108 | 497(31%) | 47.2 | 4.7 | 133(27%) | 46.6 | 9.0 | 364 | 47.5 | 2.5 |

Abbreviations: RRSO, Risk-Reducing Salpingo-oophorectomy; BC, breast cancer

^a^as a percentage of total number of individuals in each study; ^b^as a percentage of all RRSO taking place before censoring.

**Table S4. Characteristics of cohort of *BRCA1* and *BRCA2* mutation carriers**

|  | ***BRCA1* mutation carriers** | | ***BRCA2* mutation carriers** | |
| --- | --- | --- | --- | --- |
|  | Unaffected  women  (N=2,003) | Women with breast cancer  (N=269) | Unaffected  women  (N=1,448) | Women with breast cancer  (N=157) |
| Family history of breast cancer at baseline |  |  |  |  |
| No relatives with breast cancer | 545 | 54 | 284 | 17 |
| One relative with breast cancer | 628 | 91 | 445 | 49 |
| Two or more relative with breast cancer | 626 | 108 | 553 | 78 |
| Unknown family history of breast cancer | 192 | 13 | 153 | 13 |
| Cancer of unknown type in family | 12 | 3 | 13 | 0 |
| Family history of ovarian cancer at baseline |  |  |  |  |
| No relatives with ovarian cancer | 1017 | 169 | 955 | 109 |
| One relative with ovarian cancer | 538 | 58 | 265 | 29 |
| Two or more relatives with ovarian cancer | 244 | 26 | 62 | 6 |
| Parity (full-term pregnancies) |  |  |  |  |
| Nulliparous | 601 | 47 | 405 | 19 |
| 1 | 294 | 39 | 196 | 12 |
| 2 | 651 | 96 | 447 | 58 |
| 3 | 291 | 44 | 263 | 33 |
| ≥4 | 166 | 11 | 137 | 17 |
| Unknown | 0 | 32 | 0 | 18 |
| Age at first birth (years) |  |  |  |  |
| <30 | 1,057 | 157 | 785 | 95 |
| >=30 | 344 | 51 | 258 | 32 |
| Unknown | 1 | 14 | 0 | 11 |
| BMI (kg/m2) |  |  |  |  |
| <25 | 776 | 92 | 644 | 58 |
| 25-29.9 | 290 | 33 | 243 | 21 |
| >=30 | 158 | 27 | 140 | 18 |
| Unknown | 779 | 117 | 421 | 60 |
| Hormone Replacement Therapy use^a^ |  |  |  |  |
| None reported at baseline | 1,411 | 171 | 1,041 | 107 |
| HRT at baseline | 252 | 35 | 165 | 28 |
| HRT subsequent to baseline | 116 | 24 | 81 | 10 |
| Age at start of HRT use (mean (sd)) | 42.34 (6.14) | 41.8 (6.83) | 44.3 (6.38) | 45 (6.32) |

Abbreviations: BMI, Body Mass Index; Hormone Replacement Therapy, HRT

^a^ HRT use refers to any exposure, of any formulation of HRT for any length of time prior to the usual censoring age. For analyses of women without HRT, women were censored at the usual censoring age, or at the age at HRT, whichever was first. Age at start of HRT use was available for 368 *BRCA1* and 251 *BRCA2* mutation carriers.

**Table S5. Association between RRSO and breast cancer by menopausal status**

|  | ***BRCA1* mutation carriers** | | | | ***BRCA2* mutation carriers** | | | |
| --- | --- | --- | --- | --- | --- | --- | --- | --- |
|  | Person- years | BC | HR | 95%CI | Person-  years | BC | HR | 95%CI |
| Premenopausal women | | |  |  |  |  |  |  |
| No RRSO | 7499 | 140 | 1.00 | - | 4820 | 79 | 1.00 | - |
| RRSO at any age (years) | 2905 | 82 | 1.11 | 0.82 - 1.50 | 1489 | 31 | 0.69 | 0.44 - 1.08 |
| <=45 | 2076 | 59 | 1.11 | 0.80 - 1.52 | 869 | 14 | 0.57 | 0.32 - 1.01 |
| > 45 | 828 | 23 | 1.11 | 0.63 - 1.94 | 620 | 17 | 0.93 | 0.49 - 1.76 |
| Time since RRSO (years) |  |  |  |  |  |  |  |  |
| <2 | 891 | 28 | 1.18 | 0.79 - 1.76 | 524 | 14 | 0.95 | 0.54 - 1.67 |
| 2-5 | 972 | 26 | 1.04 | 0.68 - 1.59 | 541 | 11 | 0.64 | 0.34 - 1.22 |
| > 5 | 1042 | 28 | 1.09 | 0.68 - 1.75 | 424 | 6 | 0.39 | 0.16 - 0.97 |
| Postmenopausal women | | |  |  |  |  |  |  |
| No RRSO | 609 | 9 | 1.00 | - | 679 | 21 | 1.00 | -­­­­ |
| RRSO at any age | 572 | 18 | 1.69 | 0.73 - 3.91 | 330 | 10 | 1.46 | 0.66 - 3.19 |
| Time since RRSO (years) |  |  |  |  |  |  |  |  |
| <2 | 159 | 9 | 2.98 | 1.22 - 7.29 | 122 | 7 | 2.66 | 0.99 - 7.13 |
| 2-5 | 201 | 3 | 0.79 | 0.21 - 2.90 | 120 | 3 | 0.99 | 0.29 - 3.42 |
| > 5 | 211 | 6 | 1.27 | 0.39 - 4.15 | 88 | 0 | 0.00 | - |

Abbreviations: RRSO, Risk-Reducing Salpingo-oophorectomy; BC, breast cancer. A Cox regression model was used adjusting for country, stratified by year of birth (≤1960, >1960) and with robust standard errors (clustering by family). Following hysterectomy 6 BCs were diagnosed among *BRCA1* mutation carriers without RRSO and 5 BCs among *BRCA2* mutation carriers without RRSO, and 7 and 5 BCs, respectively, after RRSO.

**Table S6. Association between RRSO and breast cancer (sensitivity analysis with RRSO status changing at the age at the questionnaire with information on RRSO status changes (all studies except HEBON))**

|  | ***BRCA1* mutation carriers** | | | | | | ***BRCA2* mutation carriers** | | | | | |
| --- | --- | --- | --- | --- | --- | --- | --- | --- | --- | --- | --- | --- |
|  | Person- years | BC | HR | L95%CI | U95%CI | p-value | Person- years | BC | HR | L95%CI | U95%CI | p-value |
| **All women** | | | | | | | | | | | | |
| No RRSO | 9587 | 193 | 1.00 | - | - | - | 6447 | 124 | 1.00 | - | - | - |
| RRSO at any age | 2754 | 76 | 1.18 | 0.88 | 1.58 | 0.27 | 1439 | 33 | 0.83 | 0.54 | 1.27 | 0.39 |
| <=45 years | 1452 | 38 | 1.08 | 0.76 | 1.52 | 0.67 | 657 | 14 | 0.85 | 0.47 | 1.54 | 0.58 |
| >45 years | 1302 | 38 | 1.38 | 0.87 | 2.17 | 0.17 | 782 | 19 | 0.82 | 0.48 | 1.38 | 0.45 |
| **Time since RRSO (years)** | | | | | | | | | | | | |
| <2 | 455 | 12 | 1.07 | 0.60 | 1.91 | 0.82 | 275 | 10 | 1.38 | 0.71 | 2.69 | 0.34 |
| 2-5 | 874 | 25 | 1.20 | 0.78 | 1.85 | 0.40 | 533 | 14 | 0.92 | 0.51 | 1.64 | 0.77 |
| > 5 | 1425 | 39 | 1.21 | 0.83 | 1.76 | 0.33 | 631 | 9 | 0.51 | 0.26 | 1.03 | 0.06 |
| **Premenopausal women** | | | | | | | | | | | | |
| No RRSO | 8467 | 171 | 1.00 | - | - | - | 5317 | 90 | 1.00 | - | - | - |
| RRSO at any age | 1936 | 51 | 1.01 | 0.73 | 1.41 | 0.93 | 992 | 20 | 0.65 | 0.36 | 1.17 | 0.15 |
| <=45 years | 1340 | 33 | 0.96 | 0.67 | 1.38 | 0.84 | 573 | 11 | 0.69 | 0.35 | 1.37 | 0.29 |
| >45 years | 596 | 18 | 1.23 | 0.66 | 2.26 | 0.51 | 419 | 9 | 0.61 | 0.27 | 1.37 | 0.23 |
| **Time since RRSO (years)** | | | | | | | | | | | | |
| <2 | 374 | 8 | 0.81 | 0.40 | 1.61 | 0.54 | 212 | 6 | 1.02 | 0.43 | 2.42 | 0.97 |
| 2-5 | 669 | 19 | 1.10 | 0.68 | 1.76 | 0.71 | 399 | 9 | 0.73 | 0.35 | 1.51 | 0.39 |
| > 5 | 894 | 24 | 1.07 | 0.67 | 1.70 | 0.79 | 381 | 5 | 0.37 | 0.14 | 1.01 | 0.05 |

Abbreviations: RRSO, Risk-Reducing Salpingo-oophorectomy; BC, breast cancer. A Cox regression model was used adjusting for country, stratified by year of birth (≤1960, >1960) and with robust standard errors (clustering by family). For this analysis RRSO status was kept constant from one questionnaire to another, i.e., the time dependent variable was changed at the questionnaire age when RRSO status changed, while age at RRSO and time since RRSO were kept as reported (see Methods). Sensitivity analyses were not carried out for postmenopausal women as the numbers of women were limited.

**Table S7. Association between RRSO and breast cancer (sensitivity analysis dropping individuals with missing information at baseline)**

|  | ***BRCA1* mutation carriers** | | | | | | ***BRCA2* mutation carriers** | | | | | |
| --- | --- | --- | --- | --- | --- | --- | --- | --- | --- | --- | --- | --- |
|  | Person- years | BC | HR | L95%CI | U95%CI | p-value | Person- years | BC | HR | L95%CI | U95%CI | p-value |
| **All women** | | | | | | | | | | | | |
| No RRSO | 6960 | 122 | 1.00 | - | - | - | 5044 | 84 | 1.00 | - | - | - |
| RRSO at any age | 3316 | 94 | 1.28 | 0.95 | 1.74 | 0.11 | 1891 | 42 | 0.89 | 0.61 | 1.30 | 0.56 |
| <=45 years | 1769 | 49 | 1.21 | 0.85 | 1.72 | 0.28 | 809 | 10 | 0.58 | 0.30 | 1.14 | 0.11 |
| >45 years | 1547 | 45 | 1.44 | 0.91 | 2.27 | 0.12 | 1082 | 32 | 1.13 | 0.72 | 1.77 | 0.59 |
| **Time since RRSO (years)** | | | | | | | | | | | | |
| <2 | 940 | 35 | 1.59 | 1.09 | 2.30 | 0.01 | 608 | 19 | 1.35 | 0.82 | 2.23 | 0.24 |
| 2-5 | 1049 | 23 | 0.97 | 0.61 | 1.56 | 0.91 | 641 | 14 | 0.85 | 0.48 | 1.53 | 0.59 |
| > 5 | 1327 | 36 | 1.26 | 0.82 | 1.93 | 0.29 | 642 | 9 | 0.53 | 0.26 | 1.06 | 0.07 |
| **Premenopausal women** | | | | | | | | | | | | |
| No RRSO | 6143 | 109 | 1.00 | - | - | - | 4120 | 57 | 1.00 | - | - | - |
| RRSO at any age | 2301 | 64 | 1.14 | 0.79 | 1.62 | 0.48 | 1274 | 22 | 0.66 | 0.40 | 1.08 | 0.10 |
| <=45 years | 1641 | 44 | 1.11 | 0.76 | 1.62 | 0.59 | 719 | 7 | 0.44 | 0.20 | 0.97 | 0.04 |
| >45 years | 660 | 20 | 1.27 | 0.68 | 2.39 | 0.45 | 555 | 15 | 1.02 | 0.49 | 2.12 | 0.95 |
| **Time since RSSO (years)** | | | | | | | | | | | | |
| <2 | 722 | 23 | 1.27 | 0.81 | 1.97 | 0.29 | 439 | 9 | 0.87 | 0.43 | 1.74 | 0.69 |
| 2-5 | 771 | 19 | 1.00 | 0.60 | 1.66 | 0.99 | 463 | 8 | 0.61 | 0.29 | 1.31 | 0.21 |
| > 5 | 808 | 22 | 1.11 | 0.63 | 1.96 | 0.72 | 372 | 5 | 0.41 | 0.15 | 1.16 | 0.09 |

Abbreviations: RRSO, Risk-Reducing Salpingo-oophorectomy; BC, breast cancer. A Cox regression model was used adjusting for country, stratified by year of birth (≤1960, >1960) and with robust standard errors (clustering by family). Sensitivity analyses were not carried out for postmenopausal women as the numbers of women were limited.

**Table S8. Association between RRSO and breast cancer among *BRCA1* and *BRCA2* mutation carriers (sensitivity analysis excluding women with RRSO before baseline)**

|  | ***BRCA1* mutation carriers** | | | | | | ***BRCA2* mutation carriers** | | | | | |
| --- | --- | --- | --- | --- | --- | --- | --- | --- | --- | --- | --- | --- |
|  | Person- years | BC | HR | L95%CI | U95%CI | p-value | Person- years | BC | HR | L95%CI | U95%CI | p-value |
| **All women** | | | | | | | | | | | | |
| No RRSO | 8353 | 154 | 1.00 | - | - | - | 5769 | 106 | 1.00 | - | - | - |
| RRSO at any age | 2411 | 72 | 1.26 | 0.94 | 1.68 | 0.12 | 1487 | 34 | 0.83 | 0.55 | 1.23 | 0.35 |
| <=45 years | 1423 | 45 | 1.27 | 0.91 | 1.77 | 0.16 | 640 | 12 | 0.74 | 0.40 | 1.34 | 0.32 |
| >45 years | 987 | 27 | 1.23 | 0.76 | 1.98 | 0.41 | 847 | 22 | 0.90 | 0.54 | 1.50 | 0.68 |
| **Time since RRSO (years)** | | | | | | | | | | | | |
| <2 | 1029 | 33 | 1.28 | 0.88 | 1.87 | 0.20 | 650 | 22 | 1.28 | 0.80 | 2.03 | 0.30 |
| 2-5 | 880 | 22 | 1.07 | 0.68 | 1.69 | 0.77 | 560 | 9 | 0.55 | 0.27 | 1.10 | 0.09 |
| > 5 | 502 | 17 | 1.56 | 0.92 | 2.64 | 0.10 | 277 | 3 | 0.38 | 0.12 | 1.24 | 0.11 |
| **Premenopausal women** | | | | | | | | | | | | |
| No RRSO | 7499 | 140 | 1.00 | - | - | - | 4820 | 79 | 1.00 | - | - | - |
| RRSO at any age | 1885 | 56 | 1.15 | 0.83 | 1.59 | 0.39 | 1104 | 22 | 0.69 | 0.42 | 1.12 | 0.14 |
| <=45 years | 1384 | 43 | 1.21 | 0.86 | 1.70 | 0.28 | 626 | 11 | 0.65 | 0.35 | 1.22 | 0.18 |
| >45 years | 501 | 13 | 0.93 | 0.50 | 1.73 | 0.82 | 478 | 11 | 0.75 | 0.36 | 1.57 | 0.45 |
| **Time since RRSO (years)** | | | | | | | | | | | | |
| <2 | 832 | 24 | 1.09 | 0.70 | 1.68 | 0.71 | 490 | 13 | 0.96 | 0.54 | 1.71 | 0.90 |
| 2-5 | 697 | 19 | 1.09 | 0.68 | 1.75 | 0.71 | 420 | 6 | 0.46 | 0.20 | 1.06 | 0.07 |
| > 5 | 356 | 13 | 1.51 | 0.81 | 2.83 | 0.20 | 194 | 3 | 0.49 | 0.14 | 1.66 | 0.25 |

Abbreviations: RRSO, Risk-Reducing Salpingo-oophorectomy; BC, breast cancer. A Cox regression model was used adjusting for country, stratified by year of birth (≤1960, >1960) and with robust standard errors (clustering by family). Sensitivity analyses were not carried out for postmenopausal women as the numbers of women were limited.

**Table S9. Association between family history of breast cancer and family history of ovarian cancer and RRSO uptake**

|  | | Without RRSO | | | | With RRSO | | | | OR | | L95%CI | | U95%CI | | p-value | |
| --- | --- | --- | --- | --- | --- | --- | --- | --- | --- | --- | --- | --- | --- | --- | --- | --- | --- |
|  | | N | | % | | N | | % | |  |  |  |  |  |  |  |  |
| ***BRCA1* mutation carriers** | |  | |  | |  | |  | |  | |  | |  | |  | |
| **Breast cancer** |  | | |  | |  | |  | |  | |  | |  | |  |  |
| No relatives with breast cancer | 354 | | | 59% | | 245 | | 41% | | 1.00 | | - | | - | | - |  |
| One relative with breast cancer | 469 | | | 65% | | 250 | | 35% | | 0.82 | | 0.64 | | 1.06 | | 0.132 |  |
| Two or more relatives with breast cancer | 455 | | | 62% | | 279 | | 38% | | 0.96 | | 0.75 | | 1.22 | | 0.727 |  |
| Unknown family history of breast cancer | 148 | | | 72% | | 57 | | 28% | | 0.54 | | 0.36 | | 0.80 | | 0.002 |  |
| Cancer of unknown type in family | 10 | | | 67% | | 5 | | 33% | | 0.86 | | 0.39 | | 1.91 | | 0.714 |  |
| **­­Ovarian cancer** |  | | |  | |  | |  | |  | |  | |  | |  |  |
| No relatives with ovarian cancer | 805 | | | 68% | | 381 | | 32% | | 1.00 | | - | | - | | - |  |
| One relative with ovarian cancer | 342 | | | 57% | | 254 | | 43% | | 1.66 | | 1.33 | | 2.07 | | <0.0001 |  |
| Two or more relatives with ovarian cancer | 131 | | | 49% | | 139 | | 51% | | 2.39 | | 1.81 | | 3.17 | | <0.0001 |  |
| ***BRCA2* mutation carriers** | |  | |  | |  | |  | |  | |  | |  | |  | |
| **Breast cancer** | |  | |  | |  | |  | |  | |  | |  | |  | |
| No relatives with breast cancer | | 197 | | 65% | | 104 | | 35% | | 1.00 | | - | | - | | - | |
| One relative with breast cancer | | 356 | | 72% | | 138 | | 28% | | 0.74 | | 0.52 | | 1.05 | | 0.094 | |
| Two or more relatives with breast cancer | | 413 | | 65% | | 218 | | 35% | | 0.93 | | 0.67 | | 1.30 | | 0.678 | |
| Unknown family history of breast cancer | | 130 | | 78% | | 36 | | 22% | | 0.32 | | 0.20 | | 0.52 | | 0.000 | |
| Cancer of unknown type in family | | 12 | | 92% | | 1 | | 8% | | 0.18 | | 0.05 | | 0.72 | | 0.015 | |
| **Ovarian cancer** | |  | |  | |  | |  | |  | |  | |  | |  | |
| No relatives with ovarian cancer | | 765 | | 72% | | 299 | | 28% | | 1.00 | | - | | - | | - | |
| One relative with ovarian cancer | | 172 | | 59% | | 122 | | 41% | | 1.62 | | 1.21 | | 2.16 | | 0.001 | |
| Two or more relatives with ovarian cancer | | 29 | | 43% | | 39 | | 57% | | 2.81 | | 1.63 | | 4.86 | | 0.0002 | |

Abbreviations: RRSO, Risk Reducing Salpingo-oophorectomy. ; OR, Odds Ratio Univariate logistic regression was used to evaluate the association between family history and RRSO. Family history of breast cancer was coded as: none, one, or two or more breast cancers reported in first or second degree relatives, unknown family history of breast cancer, and cancer of unknown type in family. Women with unknown family history of breast cancer were more likely not to have reported RRSO (and not to have completed questionnaire). Family history of ovarian cancer was coded as: none, one, or two or more ovarian cancers reported in first or second degree relatives.

**Table S10. Association between parity, age at first birth, and body mass index and RRSO uptake**

|  | Without RRSO | | With RRSO | | OR | L95%CI | U95%CI | p-value |
| --- | --- | --- | --- | --- | --- | --- | --- | --- |
|  | N | % | N | % |  |  |  |  |
| ***BRCA1* mutation carriers** |  |  |  |  |  |  |  |  |
| **Parity (full-term pregnancies)** |  |  |  |  |  |  |  |  |
| Nulliparous | 531 | 82% | 117 | 18% | 1.00 | - | - | - |
| One child | 248 | 74% | 85 | 26% | 1.63 | 1.18 | 2.25 | 0.003 |
| Two children | 541 | 50% | 541 | 50% | 4.87 | 3.82 | 6.21 | <0.0001 |
| Three or more children | 100 | 56% | 77 | 44% | 4.00 | 2.60 | 6.14 | <0.0001 |
| As continuous covariate |  |  |  |  |  |  | p-trend<0.0001 | |
| **Age at first birth (years)** |  |  |  |  |  |  |  |  |
| <30 years | 667 | 55% | 547 | 45% | 1.00 | - | - | - |
| >= 30 years | 230 | 58% | 169 | 42% | 0.85 | 0.68 | 1.07 | 0.17 |
| **Body mass index (kg/m^2^)** |  |  |  |  |  |  |  |  |
| <25 | 590 | 68% | 278 | 32% | 1.00 | - | - | - |
| 25-29.9 | 205 | 63% | 118 | 37% | 1.26 | 0.96 | 1.65 | 0.1 |
| >=30 | 107 | 58% | 78 | 42% | 1.62 | 1.11 | 2.36 | 0.012 |
| As continuous covariate |  |  |  |  |  |  | p-trend=0.005 | |
| ***BRCA2* mutation carriers** |  |  |  |  |  |  |  |  |
| **Parity (full-term pregnancies)** |  |  |  |  |  |  |  |  |
| Nulliparous | 367 | 87% | 57 | 13% | 1.00 | - | - | - |
| One child | 165 | 79% | 43 | 21% | 1.70 | 1.08 | 2.68 | 0.022 |
| Two children | 475 | 59% | 326 | 41% | 4.65 | 3.40 | 6.35 | <0.0001 |
| Three or more children | 89 | 58% | 65 | 42% | 5.14 | 3.32 | 7.97 | <0.0001 |
| As continuous covariate |  |  |  |  |  |  | p-trend=<0.0001 | |
| **Age at first birth (years)** |  |  |  |  |  |  |  |  |
| <30 years | 533 | 61% | 347 | 39% | 1.00 | - | - | - |
| >= 30 years | 200 | 69% | 91 | 31% | 0.65 | 0.49 | 0.87 | 0.004 |
| **Body mass index (kg/m^2^)** |  |  |  |  |  |  |  |  |
| <25 | 508 | 72% | 194 | 28% | 1.00 | - | - | - |
| 25-29.9 | 179 | 68% | 85 | 32% | 1.31 | 0.96 | 1.78 | 0.09 |
| >=30 | 104 | 66% | 54 | 34% | 1.37 | 0.94 | 2.00 | 0.102 |
| As continuous covariate |  |  |  |  |  |  | p-trend=0.031 | |

Abbreviations: RRSO, Risk Reducing Salpingo-oophorectomy; OR, Odds Ratio. Univariate logistic regression was used to evaluate the association between, parity or age at first birth, and RRSO. Age at first birth was categorised as nulliparous, <30 and >=30 years, parity as nulliparous, 1, 2 or 3, and 4 or more children reported prior to censoring. Body Mass Index was categorised as <25, 25-29.9 and 30 kg/m^2^ or greater, reported at baseline questionnaire.

**Table S11. Association between RRSO and breast cancer adjusting for Body Mass Index, family history of breast cancer, family history of ovarian cancer, parity and age at first birth**

|  | ***BRCA1* mutation carriers** | | | | ***BRCA2* mutation carriers** | | | |
| --- | --- | --- | --- | --- | --- | --- | --- | --- |
|  | HR | L95%CI | U95%CI | p-value | HR | L95%CI | U95%CI | p-value |
| **All women** | | | | | | | | |
| No RRSO | 1.00 | - | - | - | 1.00 | - | - | - |
| RRSO All ages | 1.41 | 0.93 | 2.14 | 0.10 | 0.88 | 0.53 | 1.45 | 0.88 |
| <=45 years | 1.32 | 0.84 | 2.07 | 0.23 | 0.80 | 0.40 | 1.58 | 0.80 |
| >45 years | 1.67 | 0.89 | 3.16 | 0.11 | 0.97 | 0.48 | 1.93 | 0.97 |
| **Time since RRSO (years)** | | | | | | | | |
| <2 | 1.41 | 0.79 | 2.51 | 0.24 | 1.36 | 0.72 | 2.55 | 1.36 |
| 2-5 | 1.35 | 0.74 | 2.46 | 0.33 | 0.74 | 0.31 | 1.76 | 0.74 |
| > 5 | 1.48 | 0.84 | 2.59 | 0.18 | 0.52 | 0.20 | 1.32 | 0.52 |
| **Premenopausal women** | | | | | | | | |
| No RRSO | 1.00 | - | - | - | 1.00 | - | - | - |
| RRSO All ages | 1.15 | 0.72 | 1.84 | 0.56 | 0.60 | 0.31 | 1.13 | 0.60 |
| <=45 years | 1.12 | 0.69 | 1.83 | 0.64 | 0.59 | 0.28 | 1.27 | 0.59 |
| >45 years | 1.26 | 0.58 | 2.75 | 0.56 | 0.60 | 0.23 | 1.57 | 0.60 |
| **Time since RSSO (years)** | | | | | | | | |
| <2 y | 1.18 | 0.62 | 2.23 | 0.62 | 0.73 | 0.31 | 1.74 | 0.73 |
| 2-5 | 1.26 | 0.68 | 2.32 | 0.46 | 0.61 | 0.24 | 1.53 | 0.61 |
| > 5 y | 0.98 | 0.47 | 2.05 | 0.97 | 0.36 | 0.10 | 1.33 | 0.36 |

Abbreviations: RRSO, Risk Reducing Salpingo-oophorectomy. A Cox regression model was used adjusting for country, stratified by year of birth (≤1960, >1960) and adjusting for country, BMI, parity, age at first birth, family history of breast and family history of ovarian cancer. Family history of breast cancer was coded as: none, one or two or more breast cancers reported in first or second degree relatives. Family history of ovarian cancer was coded as: none, one or two or more ovarian cancers reported in first or second degree relatives. Age at first birth was categorised as nulliparous, <30 and >=30 years, parity as nulliparous, 1, 2 or 3, and 4 or more children reported prior to censoring. Body Mass Index was categorised as <25, 25-29.9 and 30 kg/m^2^ or greater, reported in baseline questionnaire.

**Table S12. Hormone replacement therapy use among women in the cohort**

|  | Women not diagnosed with breast cancer (N) | % | Women diagnosed with breast cancer (N) | % | Total |
| --- | --- | --- | --- | --- | --- |
| ***BRCA1* mutation carriers** |  |  |  |  |  |
| No RRSO |  |  |  |  |  |
| Not exposed to HRT^a^ | 992 | 89% | 124 | 11% | 1116 |
| Exposed to HRT^b^ | 58 | 87% | 9 | 13% | 67 |
| Exposed to HRT^c^ <=40 years | 13 | 81% | 3 | 19% | 16 |
| Information missing | 298 | 93% | 21 | 7% | 319 |
| RRSO |  |  |  |  |  |
| Not exposed to HRT | 419 | 90% | 47 | 10% | 466 |
| Exposed to HRT | 310 | 86% | 50 | 14% | 360 |
| Exposed to HRT <=40 years | 121 | 85% | 22 | 15% | 143 |
| Information missing | 66 | 79% | 18 | 21% | 84 |
| ***BRCA2* mutation carriers** |  |  |  |  |  |
| No RRSO |  |  |  |  |  |
| Not exposed to HRT | 766 | 90% | 81 | 10% | 847 |
| Exposed to HRT | 58 | 81% | 14 | 19% | 72 |
| Exposed to HRT <=40 years | 9 | 100% | 0 | 0% | 9 |
| Information missing | 213 | 95% | 11 | 5% | 224 |
| RRSO |  |  |  |  |  |
| Not exposed to HRT | 275 | 91% | 26 | 9% | 301 |
| Exposed to HRT | 188 | 89% | 24 | 11% | 212 |
| Exposed to HRT <=40 years | 51 | 89% | 6 | 11% | 57 |
| Information missing | 39 | 98% | 1 | 3% | 40 |

Abbreviations: RRSO, Risk Reducing Salpingo-oophorectomy; HRT, Hormone replacement therapy. Exposure to HRT is defined as any formulation for any duration of use. ^a^ Women not exposed to HRT at baseline questionnaire. ^b^ Women exposed to HRT at baseline questionnaire or during follow-up but before the usual censoring age. ^c^ Age at start of HRT use

**Table S13. Association between RRSO and breast cancer among women not exposed to hormone replacement therapy**

| **Category** | Person-years | BC | HR | L95%CI | U95%CI | p-value |
| --- | --- | --- | --- | --- | --- | --- |
| ***BRCA1 mutation carriers*** | | | | | | |
| No RRSO | 6630 | 124 | 1.00 | **-** | **-** | **-** |
| RRSO at any age (years) | 1777 | 47 | 1.13 | 0.77 | 1.65 | 0.54 |
| <=45 | 828 | 19 | 1.04 | 0.65 | 1.67 | 0.88 |
| >45 | 949 | 28 | 1.26 | 0.70 | 2.27 | 0.44 |
| **Time since RRSO (years)** | | | | | | |
| <2 | 594 | 15 | 1.09 | 0.62 | 1.92 | 0.76 |
| 2-5 | 585 | 15 | 1.17 | 0.65 | 2.11 | 0.60 |
| > 5 | 598 | 17 | 1.14 | 0.61 | 2.13 | 0.69 |
| ***BRCA2 mutation carriers*** | | | | | | |
| No RRSO | 4601 | 81 | 1.00 |  |  |  |
| RRSO at any age (years) | 1038 | 26 | 1.04 | 0.66 | 1.67 | 0.85 |
| <=45 | 407 | 6 | 0.64 | 0.29 | 1.42 | 0.27 |
| >45 | 630 | 20 | 1.38 | 0.77 | 2.45 | 0.28 |
| **Time since RRSO** | | | | | | |
| <2 | 397 | 15 | 1.63 | 0.91 | 2.93 | 0.10 |
| 2-5 | 386 | 8 | 0.81 | 0.38 | 1.73 | 0.59 |
| > 5 | 254 | 3 | 0.48 | 0.15 | 1.56 | 0.22 |

Abbreviations: RRSO, Risk Reducing Salpingo-oophorectomy; BC, breast cancer; HRT, Hormone replacement therapy. Analyses of the effect of RRSO on breast cancer risk, confined to women who report no hormone replacement therapy at baseline. Analyses were censored at the usual censoring age, or at any recorded age at HRT use during follow-up, whichever was first. A Cox regression model was used adjusting for country, stratified by year of birth (≤1960, >1960) and with robust standard errors (clustering by family).

**Table S14. Association between RRSO and breast cancer (excluding kConFab/BCFR)**

| **Category** | Person-years | BC | HR | L95%CI | U95%CI | p-value |
| --- | --- | --- | --- | --- | --- | --- |
| ***BRCA1 mutation carriers*** | | | | | | |
| No RRSO | 4903 | 86 | 1.00 | **-** | **-** | **-** |
| RRSO at any age (years) | 2730 | 78 | 1.17 | 0.84 | 1.61 | 0.35 |
| <=45 | 1433 | 43 | 1.13 | 0.78 | 1.62 | 0.53 |
| >45 | 1297 | 35 | 1.29 | 0.72 | 2.32 | 0.39 |
| **Time since RRSO (years)** | | | | | | |
| <2 | 806 | 32 | 1.49 | 0.99 | 2.23 | 0.06 |
| 2-5 | 892 | 18 | 0.81 | 0.49 | 1.33 | 0.40 |
| > 5 | 1033 | 28 | 1.16 | 0.72 | 1.87 | 0.53 |
| ***BRCA2 mutation carriers*** | | | | | | |
| No RRSO | 2809 | 54 | 1.00 | - | - | - |
| RRSO at any age (years) | 1319 | 32 | 0.91 | 0.58 | 1.43 | 0.68 |
| <=45 | 601 | 11 | 0.65 | 0.33 | 1.27 | 0.20 |
| >45 | 717 | 21 | 1.25 | 0.71 | 2.19 | 0.44 |
| **Time since RRSO** | | | | | | |
| <2 | 281 | 13 | 0.99 | 0.54 | 1.81 | 0.97 |
| 2-5 | 483 | 14 | 1.02 | 0.56 | 1.87 | 0.94 |
| > 5 | 355 | 5 | 0.57 | 0.21 | 1.52 | 0.26 |

Abbreviations: RRSO, Risk-Reducing Salpingo-oophorectomy; BC, breast cancer. A Cox regression model was used adjusting for country, stratified by year of birth (≤1960, >1960) and with robust standard errors (clustering by family).

**Table S15. Association between natural menopause and breast cancer (censoring at RRSO)**

|  | *BRCA1* mutation carriers | | | |  | *BRCA2* mutation carriers | | | |
| --- | --- | --- | --- | --- | --- | --- | --- | --- | --- |
|  | Person-years | BC | HR | 95%CI |  | Person-years | BC | HR | 95%CI |
| Premenopausal | 7521 | 140 | 1.00 | - |  | 4862 | 78 | 1.00 | - |
| Natural menopause at any age (years) | 609 | 9 | 0.77 | 0.31 - 1.95 |  | 679 | 21 | 1.33 | 0.57 - 3.11 |
| <=45 | 101 | 2 | 0.85 | 0.18 - 4.09 |  | 149 | 5 | 1.81 | 0.64 - 5.10 |
| > 45 | 508 | 7 | 0.75 | 0.27 - 2.07 |  | 550 | 16 | 1.15 | 0.47 - 2.80 |
| Time since natural menopause (years) | | | | | | | | | |
| <2 | 70 | 1 | 0.46 | 0.06 - 3.36 |  | 46 | 0 | 0.00 | - |
| 2-5 | 123 | 4 | 1.25 | 0.46 - 3.30 |  | 113 | 6 | 1.91 | 0.65 - 5.56 |
| > 5 y | 416 | 4 | 0.57 | 0.13 - 2.54 |  | 520 | 15 | 1.49 | 0.55 - 4.02 |

Abbreviations: RRSO, Risk Reducing Salpingo-oophorectomy; BC, breast cancer. A Cox regression model was used adjusting for country, stratified by year of birth (≤1960, ≥1960) and with robust standard errors (clustering by family)

**Ethics Committee Approvals**

GENEPSO:

A CNIL (Commission Nationale de l'Informatique et des Libertés) agreement has been obtained in February 2000 (N°999350-1999) with a last amendment in 2017 (n°999350 version 4-2017).

kConFab:

HREC approval from Peter MacCallum cancer Centre.  Ethics Ref # 97_27

BCFR:

Metropolitan New York Registry of Breast Cancer Families: Prospective Family Cancer Cohort. Columbia University Human Subjects Protocol data sheet.

Protocol number: AAAA794(M00Y26) Approved. Expiration date: 09/03/2020

EMBRACE:

East of England - Cambridge South Research Ethics Committee

IRAS project ID 20971

REC reference MREC 98/5/027 (updated 26th January 2017; substantial amendment approved 29/10/2019).

HEBON:

Approvals were non-centralized:

| **Center** | **HEBON study approval numbers** |
| --- | --- |
| Netherlands Cancer Institute (coordinating center) | PTC11.0533  PTC11.1799  PTC13.1258  IRBd19043 |
| Amsterdam UMC | 2011/253  A2016.284 |
| UMC Groningen | METc 2011/303 |
| Leiden UMC | P06.021 |
| Maastricht UMC | METC 11-4-089 |
| Radboudumc, Nijmegen | 2015-2207 |
| Erasmus MC, Rotterdam | MEC-2011-471 |
| UMC Utrecht | 11/339 |
